# Detection of characteristic sub pathway network for angiogenesis based on the comprehensive pathway network

**DOI:** 10.1186/1471-2105-11-S1-S32

**Published:** 2010-01-18

**Authors:** Yezhou Huang, Shao Li

**Affiliations:** 1 MOE Key Laboratory of Bioinformatics and Bioinformatics Div, TNLIST/Department of Automation, Tsinghua University, Beijing 100084, PR China

## Abstract

**Background:**

Pathways in biological system often cooperate with each other to function. Changes of interactions among pathways tightly associate with alterations in the properties and functions of the cell and hence alterations in the phenotype. So, the pathway interactions and especially their changes over time corresponding to specific phenotype are critical to understanding cell functions and phenotypic plasticity.

**Methods:**

With prior-defined pathways and incorporated protein-protein interaction (PPI) data, we counted PPIs between corresponding gene sets of each pair of distinct pathways to construct a comprehensive pathway network. Then we proposed a novel concept, characteristic sub pathway network (CSPN), to realize the phenotype-specific pathway interactions. By adding gene expression data regarding a given phenotype, angiogenesis, active PPIs corresponding to stimulation of interleukin-1 (IL-1) and tumor necrosis factor α (TNF-α) on human umbilical vein endothelial cells (HUVECs) respectively were derived. Two kinds of CSPN, namely the static or the dynamic CSPN, were detected by counting active PPIs.

**Results:**

A comprehensive pathway network containing 37 signalling pathways as nodes and 263 pathway interactions were obtained. Two phenotype-specific CSPNs for angiogenesis, corresponding to stimulation of IL-1 and TNF-α on HUVEC respectively, were addressed. From phenotype-specific CSPNs, a static CSPN involving interactions among B cell receptor, T cell receptor, Toll-like receptor, MAPK, VEGF, and ErbB signalling pathways, and a dynamic CSPN involving interactions among TGF-β, Wnt, p53 signalling pathways and cell cycle pathway, were detected for angiogenesis on HUVEC after stimulation of IL-1 and TNF-α respectively. We inferred that, in certain case, the static CSPN maintains related basic functions of the cells, whereas the dynamic CSPN manifests the cells' plastic responses to stimulus and therefore reflects the cells' phenotypic plasticity.

**Conclusion:**

The comprehensive pathway network helps us realize the cooperative behaviours among pathways. Moreover, two kinds of potential CSPNs found in this work, the static CSPN and the dynamic CSPN, are helpful to deeply understand the specific function of HUVEC and its phenotypic plasticity in regard to angiogenesis.

## Background

The advent of high-throughput technologies has encouraged the appearance of a lot of analysing tools, which were intended for interpreting gene expression data and extracting biological insight. No longer being limited to produce a ranked list of differentially expressed genes, most of those analysing tools are designed from the perspective of system biology and identify gene sets over-represented in biological processes [1-6]. A gene set could be a group of genes corresponding to a pathway or a group of genes sharing the same Gene Ontology term. Gene Set Enrichment Analysis [2] and other similar tools are capable to determine pathways which are enriched in the gene list of specific phenotype. Yet these methods fail to furnish the interactions among pathways, not to mention changes of the pathway interactions. Actually, the interactions among pathways are helpful for better understanding of cooperation of pathways which are critical in the functioning of each related pathway. Likewise, changes of the pathway interactions are important for comprehending alterations in the properties and functions of the cell and therefore the phenotypic plasticity, which is cell's ability to alter phenotype in response to specific environmental stimulus [1,7,8].

In recent years, several efforts have been made to surmount the limitation of those methods. Global pathway crosstalk network (GPCN) based method [5] calculates possible crosstalks among pathways and then analyzes microarray data based on these crosstalks to infer sub networks where enriched pathways tied closely to each other as potential modules. Pathway dependency structure (PDS) based method [6], in the first place, determines pathways involved in two transitions of cancer progression, and successively constructs a pathway network for each transition according to the interdependences of pathways. However, there are still problems to overcome. (i) Although whether interactions among pathways exist could be determined and p-values could be given, the types of interactions are lack in consideration and discussion. GPCN based method did not consider sharing components [5], which means that different pathways have common components and interact with each other via them, while embedded pathway network based method just considered sharing components [9]. (ii) Generally, the components and their interrelations within distinct pathways are manually collected from literature or other resources by expert curators. Consequently, the importance of context that some genes in specific pathway may be not involved under particular condition must be considered. However, GPCN based method and PDS based method both did not take into account this importance, though gene level analysis of the latter considered it. As a result, these two methods both missed interactions between pairs of pathways at least one of which was not enriched [5,6]. And GPCN based method even missed interactions which were significant in current case but insignificant in comprehensive pathway network [5]. (iii) The alteration of pathway interactions remains to be studied. GPCN based method did not consider the alteration of interactions [5]. And since PDS based method had no whole possible interdependence network as the base of analysis, nodes of networks constructed with this method were so different that a big part of the real alteration of interactions among involved pathways was still concealed [6].

Actually, from current literatures, we found that pathway may interact with one other by means of five ways: sharing components [9], components physically interacting with counterparts from the other pathway [9,10], components being enzymatic targets of the other pathway [10], components relating with components of the other pathway via transcription [10], one pathway generating signal which modulates or competes for a key modulator or mediator of the other [10]. At length, the first way means distinct pathways have common components like genes or gene products and these shared components produce interactions among pathways; the second way depends on the physical protein-protein interactions (PPIs) like binding and phosphorylation; the third way could be reflected by the indirect PPI by two successive enzymes in the metabolic pathway; the fourth way could be represented by indirect PPIs between transcriptional factors and transcribed gene products, which reflect the corresponding gene regulation; the fifth way involves all kinds of PPIs mentioned above.

So we incorporated distinct PPIs to derive interactions among pathways. By counting PPIs between corresponding gene sets of each pair of pathways, pathway interactions could be assessed. Furthermore, since no gene is involved in specific pathway in any case and no PPI works under any condition, we incorporated gene expression data to infer active PPIs, which have at least one differentially expressed corresponding gene, in certain case for distinct phenotype, such as angiogenesis. And then we constructed phenotype-specific pathway networks with the derived sets of active PPIs. Two sub phenotype-specific pathway networks were detected as angiogenesis-related characteristic sub pathway networks (CSPNs) to manifest the specific function of the cell and its phenotypic plasticity with regard to angiogenesis.

Angiogenesis studied in this work, as the process of new blood vessel formation, is fundamental to reproduction, development, and repair. Strictly regulated by many inducers and inhibitors, it could be aroused when the local equilibrium between them is changed. At the phenotype level, angiogenesis is critical to inflammatory responses and cancers [11]. In our work, two series of gene expression data were used to infer CSPNs for angiogenesis. Those data are about human umbilical vein endothelial cells (HUVEC) after stimulations of Interleukin-1 (IL-1) and tumor necrosis factor α (TNF-α), both are known proangiogenic factors. Actually, after being treated with different proangiogenic factors or same factor but with various dosages, some pathway interactions of the cell keep constant, others alter. Those constant interactions are likely required immediately and continually by the cell for basic function in regard to particular stimulus like IL-1 and TNF-α for angiogenesis, whereas those changed interactions are probably reflect the phenotype plasticity concerning specific stimulus such as IL-1 and TNF-α for angiogenesis.

## Methods

### Preparation of PPI data

To complement substantial information loss of restricting the pathway interactions to those by means of physical PPIs like binding or phosphorylation, we considered all the five kinds of pathway interactions. We gathered the PPI data from Human Protein Reference Database [12] and Kyoto Encyclopedia of Genes and Genomes [13]. HPRD PPIs are mainly physical PPIs. The generalized PPI in KEGG includes three types of interactions: PPI by two successive enzymes in a known pathway, PPI like binding or phosphorylation, PPI between transcription factor and transcribed gene product via gene expression, which actually reflects the gene expression regulation between transcription factor and its regulated gene [13].

### Preparation of pathway data

Generally, established pathways from various databases can be approximately divided into three categories: metabolic pathways, signalling pathways, and pathways of specific diseases or drugs [9]. At length, metabolic pathways focus on the metabolism and metabolic products of cells, having much less relations with PPI. In contrast, signalling pathways mainly involve genes and gene products. Also, the environmental information processing is of biological essence to cells, while the cellular information processing is of clinical consequence: a breakdown in it underlies many diseases [14]. In KEGG, pathways designed for the study of specific diseases are often integrated from some other pathways in former two categories, and pathways from 'Drug development' category are drug structure maps on the structure relationships of drugs. Thus, considering the ways in which pathway interacts with each other are of proteins, we gathered 48 human pathways from 'Environmental Information Processing' and 'Cellular Processes' categories of KEGG as the reference of pathways to elucidate the cooperation of pathways in cells and cells' phenotypic plasticity.

### Construction of pathway network and detection of CSPNs

**Figure 1:**
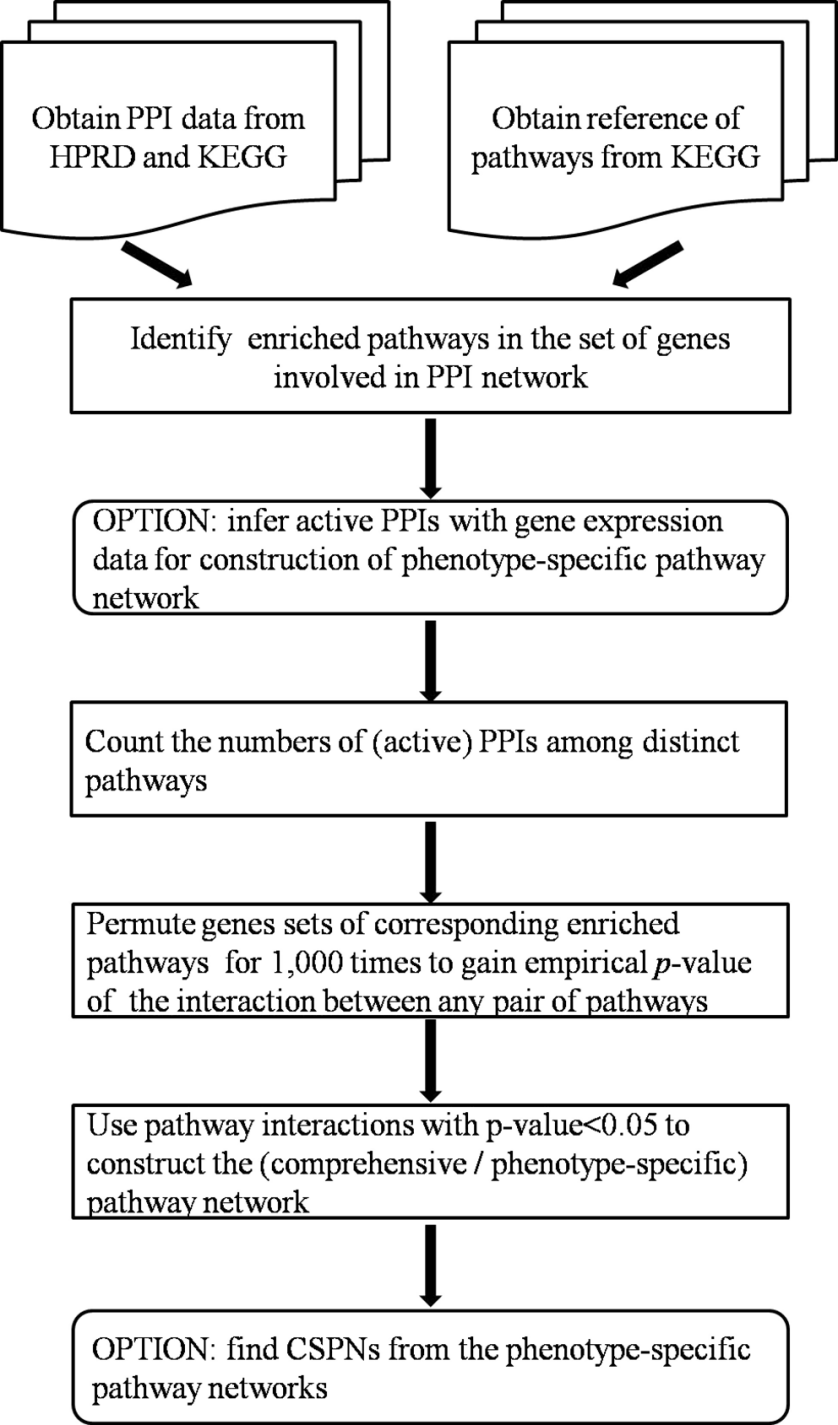
**Workflow of construction of comprehensive pathway network and detection of characteristic sub pathway networks**.

The workflow for constructing pathway network was illustrated in Figure 1 and depicted as follows. **(i) **Generate the PPI network with 57092 interactions among 10204 proteins, which are gathered from HPRD and KEGG. **(ii) **Generate the reference of pathways from 'Environmental Information Processing' and 'Cellular Processes' categories of KEGG Pathway Maps. **(iii) **Exploiting GeneMerge method [15], produce the pathways set by identifying pathways, which were enriched in the involved genes list of the PPI network from the reference of pathways. **(iv) **Option: incorporate gene expression data to infer active PPIs. Under certain condition, only a part of the PPIs could be regarded as active interactions. We obtain the set of active PPIs with Microarray data. Then, the phenotype-specific pathway network could be constructed. **(v) **Count (active) PPIs between each pair of distinct pathways. A common gene between any two pathways will be treated as two discrete genes, which interact with same genes as that original common gene, belonging to these two pathways respectively. But the relationship of sharing itself is not considered as interaction. **(vi)**Assess the effect of PPI network itself as background to the number of PPIs between any pair of pathways. Firstly, those enriched pathways are permuted by randomly exchanging two distinct genes from two different pathways for 100,000 times. Both exchanged genes should interact with at least one gene. Secondly, perform step (iv) to count PPIs between each pair of pathways after permutation. This whole pipeline is repeated for 1,000 times. And then the background distribution of the number of PPIs between each pair of pathways is obtained. Additionally, there is a second way to permute pathways: just exchange pairs of genes which have the similar number of PPIs. And the results of these two approaches in step (iv) are approximately same. **(vii) **Determine which of the pathway interactions exist by calculating the corresponding empirical p-value. For interaction between any pair of pathways, firstly, count the number of permutations after which the PPIs between the pair of permuted pathways is higher than or equal to that of the pair of real pathways. Secondly, divide the number by 1,000 which is the total number of permutations and obtain the empirical *p*-value of that interaction. All interactions with *p *< 0.05 are used to construct the pathway network. **(viii) **Option: detect CSPNs from phenotype-specific pathway networks. To detect static CSPN, we firstly identify the common static sub pathway network where nodes and edges appear at each time point of each network, then identify highly connected pathways in this sub pathway network. Along with prior biological knowledge and the degree distribution of pathways in comprehensive pathway network, part of these highly connected pathways and their interactions could be detected as static CSPN. To detect dynamic CSPN, at first, find pathways among which interactions alter obviously, then, from found pathways, further discover pathways which have relatively low degrees in comprehensive pathway network. Along with prior biological knowledge, part of these discovered pathways and their interactions could be viewed as dynamic CSPN.

### Preparation of time course Microarray data

Since angiogenesis could be aroused by different stimulus, the CSPNs for angiogenesis would be more credible if more data about different stimulus were used. Finally, two series of time course gene expression data, GSE973 and GSE9055, about human umbilical vein endothelial cells (HUVEC) after stimulations of IL-1 and TNF-α were obtained from the Gene Expression Omnibus (GEO). GSE973 [16] has four samples: HUVEC were stimulated with IL-1 for 0, 0.5, 1, 2.5 and 6 hours; and GSE9055 [17] has twenty-five samples: Using TNF-α, samples were collected every 15 min to arrays. We selected samples of 0 h 15 m, 1 h 15 m, 3 h 15 m, 8 h 00 m of GSE9055 in order to compare these two series appropriately. At each time point of these two series, only one sample (microarray) is offered. Consequently, we could not directly make use of coexpression to infer active PPI at teach time point. Also, t test and Bayesian analysis are not appropriate for discovering differentially expressed genes. Finally, the differentially expressed genes were obtained with fold change method. Taking the quality of gene expression data and the shortage of fold change method itself into account, we should admit that genes with high expression level but relatively small fold change may also reflect significant biological meaning, even if their fold change values are smaller than 2. Hence, we inferred that all the immediate neighbours of those differentially expressed genes in the PPI network were underlying relevant genes, for their approved interactions with those differentially expressed genes support the presumption of their relevance to some extent. Then we considered those PPIs which have at least one differentially expressed corresponding gene as the active PPIs. Successively, we exploited this kind of active interaction in construction of phenotype-specific pathway network.

## Results

### Comprehensive pathway network construction

We first collected 48 pathways from 'Environmental Information Processing' and 'Cellular Processes' categories of KEGG Pathway Maps. We identified 43 pathways which show significant enrichment in the list of 10204 genes involved in the generated PPI network. To some extent, these 43 pathways have relatively high ratio of proteins which have at least one interaction with other proteins, which could explain why they are enriched in the genes set of PPI network. Then a comprehensive pathway network is constructed (Table 1 and Additional file 1: Comprehensive pathway network) with 37 nodes and 263 edges. Six pathways were not represented in this network for their relatively higher independence in comprehensive condition. It was possible that the PPIs between these six pathways and other pathways were relatively less in comprehensive condition but more in specific case, or PPIs involved in these six pathways mainly were inner-pathway PPIs.

**Table 1:** The top 16 Hub pathways (degree > 18) identified in Comprehensive pathway network

Abbreviation	KEGG ID	Pathway Name	Degree
B cell	has04662	B cell receptor signaling pathway	26
ErbB	hsa04012	ErbB signaling pathway	26
FcERI	hsa04664	Fc epsilon RI signaling pathway	26
VEGF	hsa04370	VEGF signaling pathway	26
FA	hsa04510	Focal adhesion	24
GnRH	has04912	GnRH signaling pathway	24
Insulin	hsa04910	Insulin signaling pathway	23
T cell	hsa04660	T cell receptor signaling pathway	23
LtD	hsa04720	Long-term potentiation	22
Pho	hsa04070	Phosphatidylinositol signaling system	21
LtP	hsa04730	Long-term depression	20
NKCMC	hsa04650	Natural killer cell mediated cytotoxicity	20
ROAC	hsa04810	Regulation of actin cytoskeleton	20
GJ	hsa04540	Gap junction	19
MAPK	hsa04010	MAPK signaling pathway	19
mTOR	hsa04150	mTOR signaling pathway	19

### Types of pathway interaction

To understand the cooperation of distinct pathways more fully, all the five kinds of pathway interactions were considered to form pathway networks (Table 2). We made comparison between our perspective and other two perspectives, which are only considering sharing components, and only considering interactions excluding sharing components. In fact, when we only considered sharing components instead all five kinds of interactions as the origin of crosstalk, the average degree dropped. 76 interactions among 31 pathways were removed, though 23 interactions involving 18 pathways emerged. Interactions we lost are much more than interactions came forth. When we considered interactions excluding sharing components as the stem of crosstalk, the average degree of pathway network dropped observably and there were only 27 interactions regarding 18 pathways.

**Table 2:** Comparison of degrees of different pathway networks

Pathway Network	Number of Nodes	Number of Edges	Average Degree
Comprehensive pathway network based on all types of pathway interactions	37	263	7.1
Comprehensive pathway network only based on sharing components	39	210	5.4
Comprehensive pathway network based on interactions without sharing components	18	27	1.5
Phenotype-specific pathway network in IL-1 case for angiogenesis, based on comprehensive pathway network	36	222	6.2
Phenotype-specific pathway network in TNF-α case for angiogenesis, based on comprehensive pathway network	41	241	5.9
Phenotype-specific pathway network in IL-1 case for angiogenesis, based on enriched pathway pipeline	10	15	1.5

### Phenotype-specific pathway network construction

**Figure 2:**
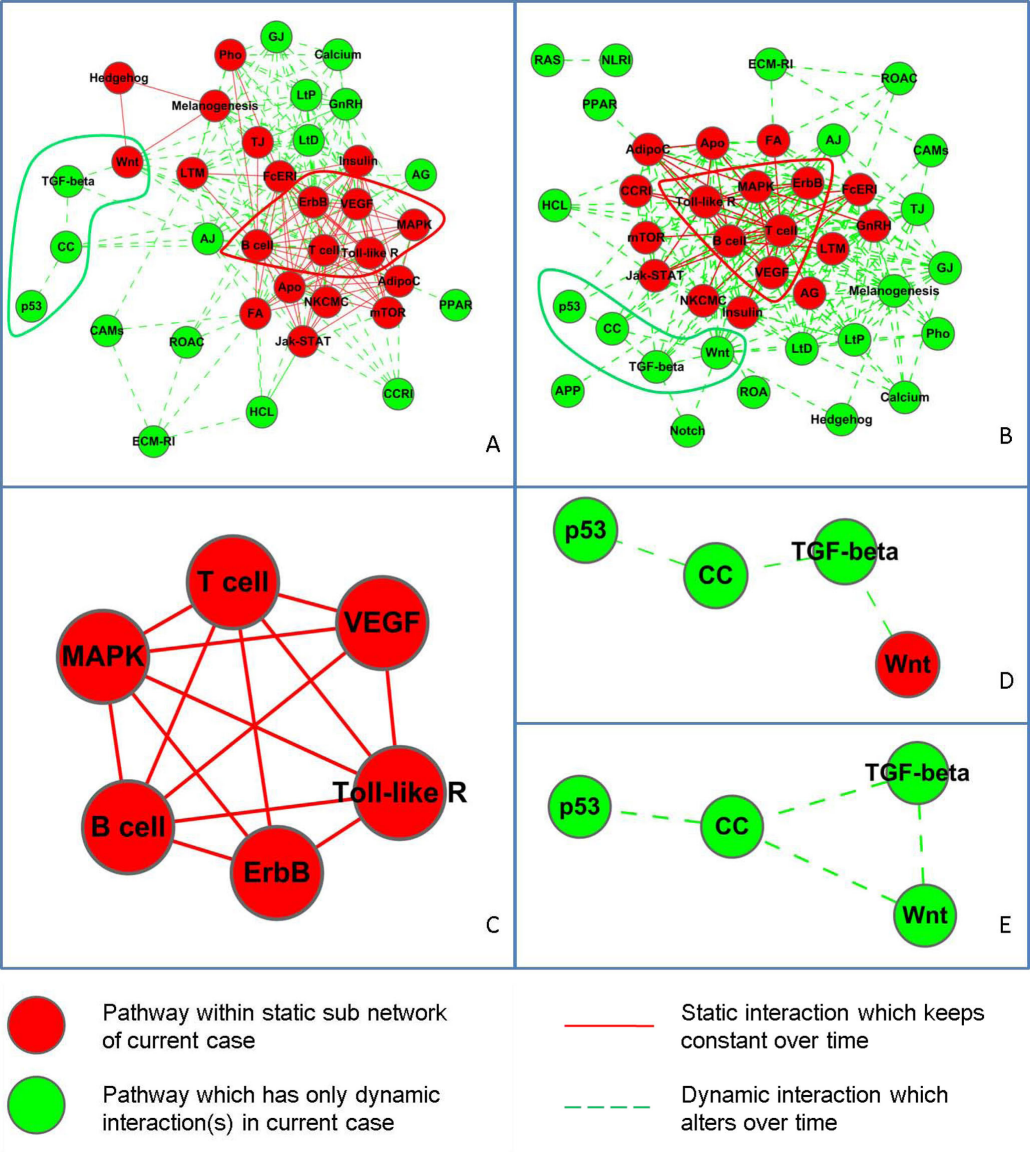
**Phenotype (angiogenesis)-specific pathway network and characteristic sub pathway networks (CSPN)**. A: Phenotype-specific pathway network in IL-1 case. B: Phenotype-specific pathway network in TNF-α case. C: Static CSPN. D: Dynamic CSPN in IL-1 case. E: Dynamic CSPN in TNF-α case.

Using gene expression data of GSE973 and GSE9055, we inferred relevant gene sets at each time points of both series, and got two phenotype-specific pathway networks for angiogenesis (Figure 2). One was of IL-1, and the other was of TNF-α. Because no PPI is active in any case, no pathway interaction always exists. Naturally, interactions in both phenotype-specific pathway networks were fewer than those in comprehensive pathway network (Table 2) and some interactions altered over time. The sub pathway network in IL-1 case, interactions within which were constant over time, was approximately identical with its counterpart in TNF-α case. A group of pathways in IL-1 case, interactions among which altered obviously over time, was nearly the same with its counterpart in TNF-α case too, though the alterations of pathway interactions in two cases were not identical.

### Quality of pathway interaction in phenotype-specific pathway network

If we take into account the interactions among pathways from a narrow perspective of enriched pathways, we will miss important interactions. There are two typical enriched pathway based approaches, both of which have this problem [5,6]. Therefore, we argue that it is more appropriate to construct pathway network for relevant case through assessing the significance of pathway interaction, no matter whether the pathways are enriched in current case or not. Actually, this approach is much better for comprehending the cooperation between distinct pathways, since the enrichment analysis only reveals to which degree the whole but not the relevant part of gene set of pathway takes part in current case. We built a phenotype-specific pathway network in IL-1 case based on enriched pathway pipeline to illustrate its shortcoming (Table 2). There were only 15 interactions involving 10 pathways in this network. Important interactions such as interaction between Wnt and TGF-β signalling pathways disappeared.

### Detecting CSPNs for angiogenesis

From two constructed phenotype-specific pathway networks, static CSPN and dynamic CSPN for angiogenesis were detected. On the one hand, firstly, the common static sub pathway network within which interactions keep constant were identified. Then a part of this sub network involving B cell receptor, T cell receptor, Toll-like receptor, MAPK, VEGF and ErbB pathways was detected as static CSPN (Figure 2). The high degrees of those pathways and the almost full connected nature of this part of network were maintained at each time point in both cases. These pathways are highly connected in comprehensive pathway network too, which means their wide and deep involvement in different phenotypes. Also, B cell receptor, T cell receptor and Toll-like receptor signalling pathways are important in immunity; MAPK pathway is involved in various cellular functions; VEGF pathway is considered to be crucial in signal transduction in both physiologic and pathologic angiogenesis; ErbB pathway couples binding of extracellular growth factor ligands to intracellular signalling pathways regulating diverse biologic responses [13,18]. Therefore, we derived that, conforming to interactions in the static CSPN, pathways in static CSPN cooperated with each other to receive, process and relay extracellular stimulus for angiogenesis. On the other hand, there was another group of pathways including TGF-β, Wnt, p53 pathways and cell cycle pathway, with interactions among them was detected as dynamic CSPN (Figure 2). Actually, those interactions changed observably over time in both series. Their relatively low degrees in comprehensive pathway interaction network might mean that they only involved in specific phenotypes. In addition, these four pathways all take part in the control of cell fate [13,19]. We inferred that the cooperation of them played a key role in manifesting response of HUVEC to stimulus like IL-1 and TNF-α via controlling development of HUVEC, and reflected the phenotypic plasticity of HUVEC in regard to angiogenesis.

In detail, interactions among pathways within static CSPN are the immediate and constant response of HUVEC to the stimulation of IL-1 or TNF-α signal. The B cell receptor, T cell receptor and Toll-like receptor signalling pathways represented the innate immune system to depress the excitement from stimulus [20]. They cooperated with each other to interfere with the interactions among MAPK, ErbB and VEGF pathways, which likely promoted angiogenesis [18,21,22]. Also, within dynamic CSPN, p53 pathway was possibly aroused to prevent angiogenesis via negatively regulating cell cycle pathway [23]. But, cooperating with each other to interact with cell cycle pathway, TGF-β and Wnt pathways inside dynamic CSPN also were stirred up to promote angiogenesis [24,25].

We compared the dynamic CSPN at each time point too (Additional file 2: Comparison of dynamic CSPN at different time points). In IL-1 case, p53 only interacted with cell cycle pathway at second and fourth time points. The first interaction might respond to the excitement from static CSPN, since MAPK, ErbB and VEGF pathway immediately cooperated with each other to promote angiogenesis after treatment. The fourth interaction probably reacted to the agitation from dynamic CSPN itself, for TGF-β and Wnt pathways collaborated to propel angiogenesis after the spread of signals from static CSPN. Actually, Wnt pathway interacted with VEGF pathway at the third time point (Additional file 3: Pathway interaction at different time point(s) in IL-1 case), which likely invoked the collaboration between Wnt and TGF-β pathways as well as their regulation to cell cycle pathway. In TNF-α case, at last three time points, p53 all interacted with cell cycle pathway, as well as TGF-β and Wnt pathways cooperated to propel angiogenesis by interacting with cell cycle pathway (see Additional file 4). Virtually, all the interactions between pathways within dynamic CSPN and pathways from static CSPN were appeared after the first time point, which possibly implied the transmission of signals from static CSPN [23].

We inferred that the difference of evolvement of dynamic CSPN between IL-1 case and TNF-α case probably reflected the diversity of traits or dosages of stimulus, though uncertainty of the cause remained before more reliable data could be gathered for this pipeline. Also, there was cooperation between static and dynamic CSPNs. The static CSPN was likely the core one, because of its immediate and sustained response to stimulus, while the dynamic CSPN was probably the complementary one, by reason of its same effect to stimulus as the static CSPN as well as the delay of its response. It is reported that crosstalk of VEGF and Notch pathways is crucial to tumor angiogenesis [26], resulting in blockade of VEGF pathway sometimes doesn't work [27]. In angiogenesis of HUVEC, it is also possible that blocking VEGF pathway make no effect for prevention of angiogenesis, on account of the work of TGF-β and Wnt pathways in dynamic CSPN.

## Discussion

In this work, we mainly proposed a methodology to consider the alteration of interactions among relevant pathways for specific phenotype, and then to detect two kinds CSPNs from constructed phenotype specific pathway networks of different cases for the same certain phenotype. We took angiogenesis on HUVEC as an example. In the past years, many useful methods, which could identify gene sets over-represented in biological processes, were offered [2,3]. For example, LMMA reconstructed the gene association network specific for angiogenesis, and extracted significant pathways from the network [3]. For moving forward to the next stage, the interactions among pathways and the alterations of these interactions require further investigation. Recently, GPCN based [5] and PDS based [6] methods were proposed to follow up this subject. But three problems still existed: the types of interactions were lack in consideration and discussion; the importance of context needed to be taken into account; the alteration of pathway interactions remained to be studied.

To solve these problems, we considered all five types of pathway interactions to construct a comprehensive pathway network, and inferred relevant genes of each pathway and active PPIs with Microarray data. Then, phenotype-specific pathway networks were constructed. From these networks, alteration of pathway interactions over time was distinct in each network, and alteration of pathway interactions with different stimulus was clear after comparison of corresponding networks. A static CSPN and a dynamic CSPN were also detected from these phenotype-specific pathway networks. The effect and cooperating mechanism of distinct pathways within both CSPNs, the influence of the diversity of traits or dosages of stimulus to the evolvement of dynamic CSPN, as well as the complementary role of dynamic CSPN to static CSPN, were elaborated.

Actually, it will enhance the reliability of "active PPI" by requiring corresponding genes firstly are coexpressed within distinct case (series in GEO). We will go further in the future to refine our work from this respect. In addition, the detection of CSPNs for specific phenotype requires ruling out the effect of other factors such as the difference of stimulus and experimental bias. As a result, the quantity and quality of corresponding time-course microarray data are asked to be appropriate to obtain reliable CSPNs. But now even the demand of quantity can not be met. We also will go further in the future to provide related active PPIs for each pathway interaction and then predict the pathway interaction type.

## Conclusion

The comprehensive pathway network helps us realize the cooperative behaviour among pathways by providing most of possible interactions among pathways. By combining phenotype-specific gene expression data, two kinds of potential CSPNs detected in this work, the static CSPN and the dynamic CSPN, are helpful to deeply understand the specific function and phenotypic plasticity of angiogenesis.

## Competing interests

The authors declare that they have no competing interests.

## Authors' contributions

Y. Huang performed the computational works. S. Li conceived and designed the research. Y. Huang and S. Li analyzed results drafted the manuscript.

## Supplementary Material

Additional file 1 **Comprehensive pathway network**. Red nodes: hub pathways. Click here for file

Additional file 2 **Comparison of dynamic CSPN at different time points**. A: Dynamic CSPN at 0.5 hour in IL-1 case. B: Dynamic CSPN at 1 hour in IL-1 case. C: Dynamic CSPN at 2.5 hours in IL-1 case. D: Dynamic CSPN at 6 hours in IL-1 case. E: Dynamic CSPN at 0 h 15 min in TNF-α case. F: Dynamic CSPN at 1 h 15 min in TNF-α case. G: Dynamic CSPN at 3 h 15 min in TNF-α case. H: Dynamic CSPN at 8 h 15 min in TNF-α case. Click here for file

Additional file 3 **Pathway interaction at different time point(s) in IL-1 case**. First and second columns are end nodes (pathways) of corresponding edge (pathway interaction). Third column is the time points at which interaction exists. Click here for file

Additional file 4 **Pathway interaction at different time point(s) in TNF-α case**. First and second columns are end nodes (pathways) of corresponding edge (pathway interaction). Third column is the time points at which interaction exists. Click here for file
