# Supplementary figures and images for: Detection of characteristic sub pathway network for angiogenesis based on the comprehensive pathway network
Source: BMC Bioinformatics. 2010 Jan 18;11(Suppl 1):S32. doi: 10.1186/1471-2105-11-S1-S32 (PMC3009504; doi:10.1186/1471-2105-11-S1-S32)

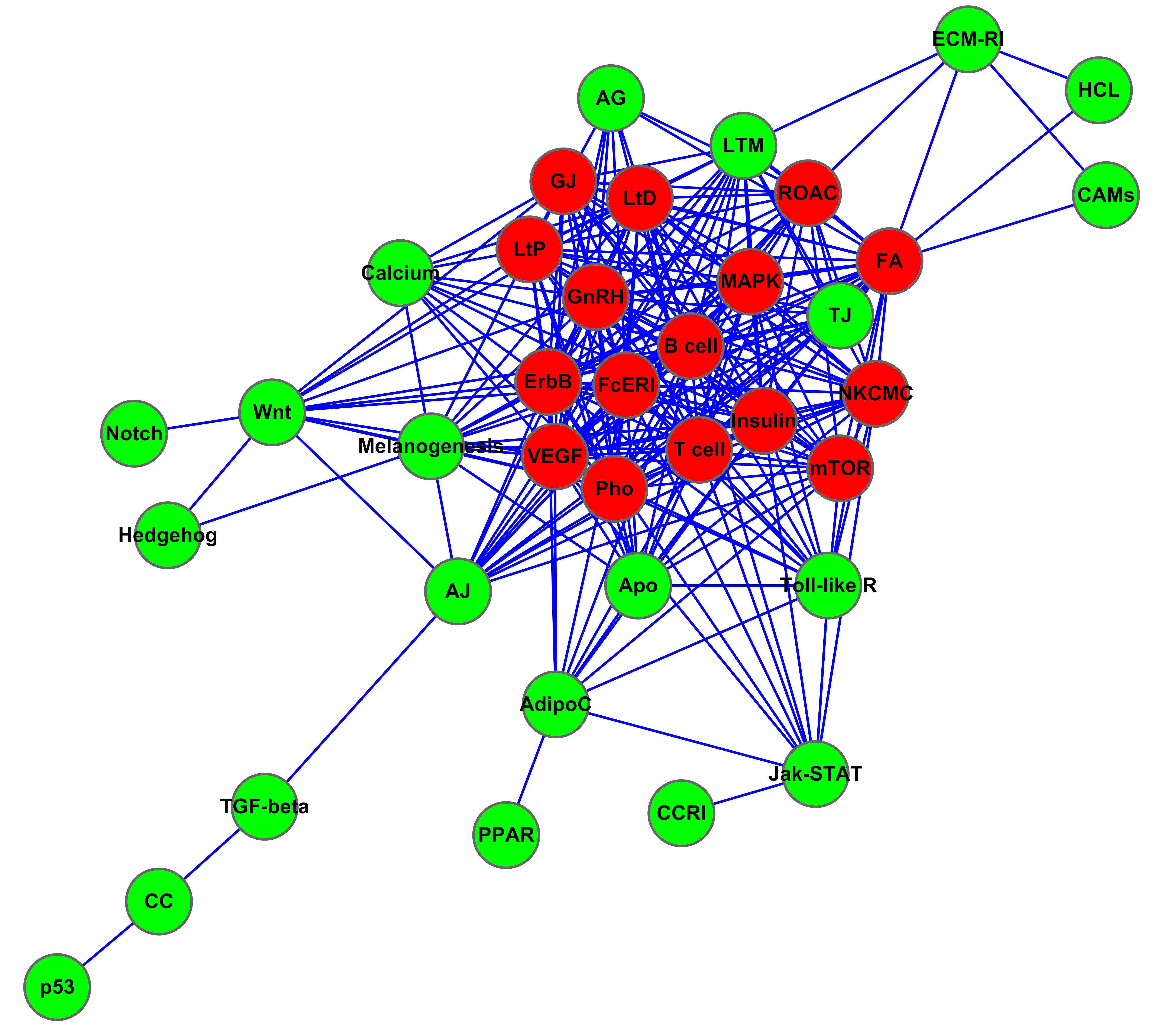

Supplement: Additional file 1 — Comprehensive pathway network. Red nodes: hub pathways. [file 1471-2105-11-S1-S32-S1.jpg]

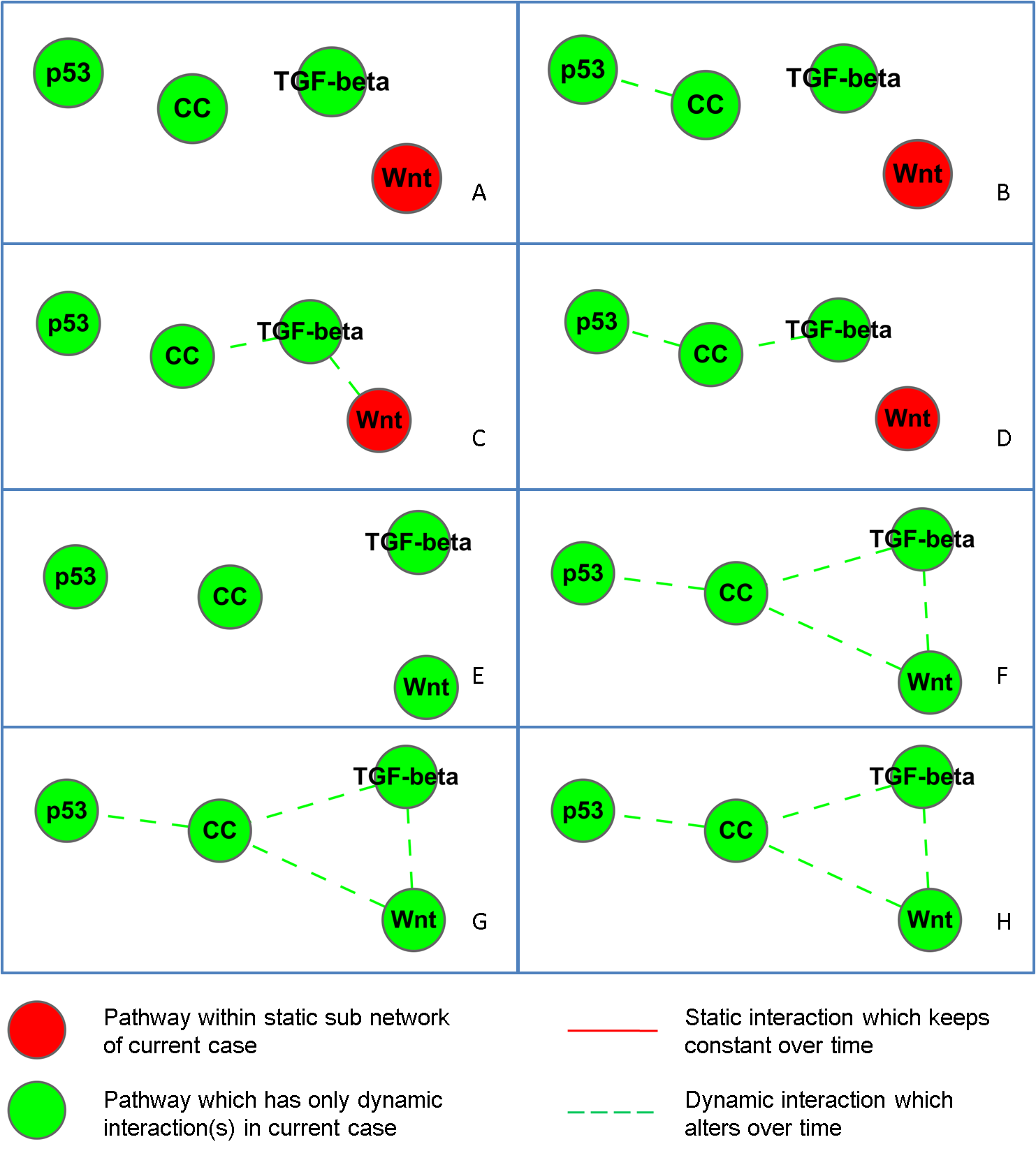

Supplement: Additional file 2 — Comparison of dynamic CSPN at different time points. A: Dynamic CSPN at 0.5 hour in IL-1 case. B: Dynamic CSPN at 1 hour in IL-1 case. C: Dynamic CSPN at 2.5 hours in IL-1 case. D: Dynamic CSPN at 6 hours in IL-1 case. E: Dynamic CSPN at 0 h 15 min in TNF-α case. F: Dynamic CSPN at 1 h 15 min in TNF-α case. G: Dynamic CSPN at 3 h 15 min in TNF-α case. H: Dynamic CSPN at 8 h 15 min in TNF-α case. [file 1471-2105-11-S1-S32-S2.png]
